# Parenteral anticoagulation may prolong the survival of patients with limited small cell lung cancer: a Cochrane systematic review

**DOI:** 10.1186/1756-9966-27-4

**Published:** 2008-05-15

**Authors:** Elie A Akl, Frederiek F van Doormaal, Maddalena Barba, Ganesh Kamath, Seo Young Kim, Saskia Kuipers, Saskia Middeldorp, Victor Yosuico, Heather O Dickinson, Holger J Schünemann

**Affiliations:** 1 Department of Medicine, State University of New York at Buffalo, NY, USA; 2 Department of Vascular Medicine, Academic Medical Centre, Amsterdam, The Netherlands; 3 Department of Epidemiology, Italian National Cancer Institute Regina Elena, Rome, Italy; 4 Department of Clinical Epidemiology, Leiden University Medical Center, The Netherlands; 5 National Guideline Research & Development Unit, University of Newcastle, UK

## Abstract

**Background:**

To determine the efficacy and safety of heparin (unfractionated heparin (UFH) or low-molecular-weight-heparin (LMWH)) and fondaparinux in improving the survival of patients with cancer.

**Methods:**

We conducted in January 2007 a comprehensive search for relevant randomized clinical trials (RCTs). We used a standardized form to extract in duplicate data on methodological quality, participants, interventions and outcomes of interest including all cause mortality, thromboembolic events, and bleeding events. We assessed the methodological quality for each outcome by grading the quality of evidence using the Grading of Recommendations Assessment, Development and Evaluation (GRADE) methodology

**Results:**

Of 3986 identified citations, we included 5 RCTs, none of which evaluated fondaparinux. The quality of evidence was moderate for survival, low for major and minor bleeding, and very low for DVT. Heparin therapy was associated with a statistically and clinically significant survival benefit (hazard ratio (HR) = 0.77; 95%CI = 0.65–0.91). In subgroup analyses, patients with limited small cell lung cancer experienced a clear survival benefit (HR = 0.56; 95%CI = 0.38–0.83). The survival benefit was not statistically significant for either patients with extensive small cell lung cancer (HR = 0.80; 95%CI = 0.60–1.06) or patients with advanced cancer (HR = 0.84; 95%CI = 0.68–1.03). The increased risk of bleeding with heparin was not statistically significant (relative risk (RR) = 1.78; 95%CI = 0.73–4.38).

**Conclusion:**

This review suggests a survival benefit of heparin in cancer patients in general, and in patients with limited small cell lung cancer in particular.

## Background

Researchers have hypothesized that heparin improves outcomes in cancer patients through an antitumor effect in addition to its antithrombotic effect [1]. In a 1992 trial comparing nadroparin, a low-molecular-weight-heparin (LMWH), to unfractionated heparin (UFH) in patients with deep vein thrombosis (DVT), nadroparin unexpectedly reduced mortality in the subgroup of cancer patients [2]. At the same time, the risk of bleeding with anticoagulants is higher in patients with cancer compared to those without cancer [3]. Heparins are also known to cause thrombocytopenia [4].

A 1999 systematic review of the effects of UFH on survival in patients with malignancy found three trials of high methodological quality but with conflicting results. [5] Since then reports on several randomized controlled trials (RCTs) on this subject have been published [6,7], including at least one study in patients with small cell lung cancer [8]. The purpose of this study was to determine the efficacy and safety of parenteral anticoagulation in improving survival of patients with cancer in general and lung cancer in particular.

## Methods

### Data Sources and Searches

The search was part of a comprehensive search for studies of anticoagulation in patients with cancer. We electronically searched in January 2007 the following databases from the date of their inception: The Cochrane Central Register of Controlled Trials, MEDLINE, EMBASE and ISI the Web of Science (see Additional file 1). We also hand searched the conference proceedings of the American Society of Clinical Oncology and of the American Society of Hematology. We reviewed the reference lists of included papers and used the related article feature in PubMed. We applied no language restrictions.

### Study Selection

Two reviewers independently screened the titles and abstracts for eligibility. We retrieved the full texts of articles judged as potentially eligible by at least one reviewer. Two reviewers then independently screened the full texts articles for eligibility and resolved their disagreements by discussion. We included abstracts only if authors supplied us with the necessary information about their methods and results.

We included only RCTs. Study participants had to have cancer but no indication for prophylactic or therapeutic anticoagulation. Interventions included one of the three classes of parenteral anticoagulants approved for clinical use: UFH, LMWH, and/or fondaparinux. The review outcomes were: survival (primary outcome), symptomatic DVT, symptomatic pulmonary embolism, major bleeding, minor bleeding, and thrombocytopenia. DVT and PE events had to be diagnosed using objective diagnostic tests.

### Data Extraction and Quality Assessment

Two reviewers independently extracted data using a standardized form and resolved their disagreements by discussion. We contacted authors for incompletely reported data.

We extracted time to event data by abstracting the log(hazard ratio) and its variance from trial reports; if these were not reported, we digitised the published Kaplan-Meier survival curves and estimated the log(hazard ratio) and its variance using Parmar's methods [9]. We performed these calculations in Stata 9, using a specially written program, which yielded the reported log(HR) and variance when used on the data presented in Table V of Parmar 1998 [9].

We also extracted categorical data necessary to conduct intention-to-treat (ITT) analyses. We collected all cause mortality at one year (time point defined a priori) and at 2 years (time point defined post hoc based upon results reported in the individual RCTs).

We assessed the following methodological criteria: allocation concealment, blinding (patient, provider, outcome assessor, data analyst), whether the analysis followed the ITT principle, whether study was stopped early for benefit, and percentage of follow-up. We assessed the methodological quality for each outcome by grading the quality of evidence using the Grading of Recommendations Assessment, Development and Evaluation (GRADE) methodology [10].

### Analysis

We calculated the agreement between the two reviewers for eligibility assessment using kappa statistic. We created an inverted funnel plot for the primary outcome to check for possible publication bias.

For time to event data, we pooled the log(HR)s using a random-effects model and the generic inverse variance facility of RevMan 4.2. For categorical data, we calculated the relative risk (RR) separately for each study for the incidence of outcomes by treatment arm. We then pooled the results of the different studies using a random-effects model.

We measured homogeneity across studies using the I^2 ^statistics [11] and considered the following classification of heterogeneity based on the value of I^2 ^(Higgins, personal communication): 0–50 = low; 30–80 = moderate and worthy of investigation; 60–100 = severe and worthy of understanding; 95–100 = aggregate with major caution. We planned to explore heterogeneity by conducting subgroup analyses based on the type of intervention and the characteristics of participants. We also planned for sensitivity analysis excluding poor quality trials.

## Results

**Figure 1:**
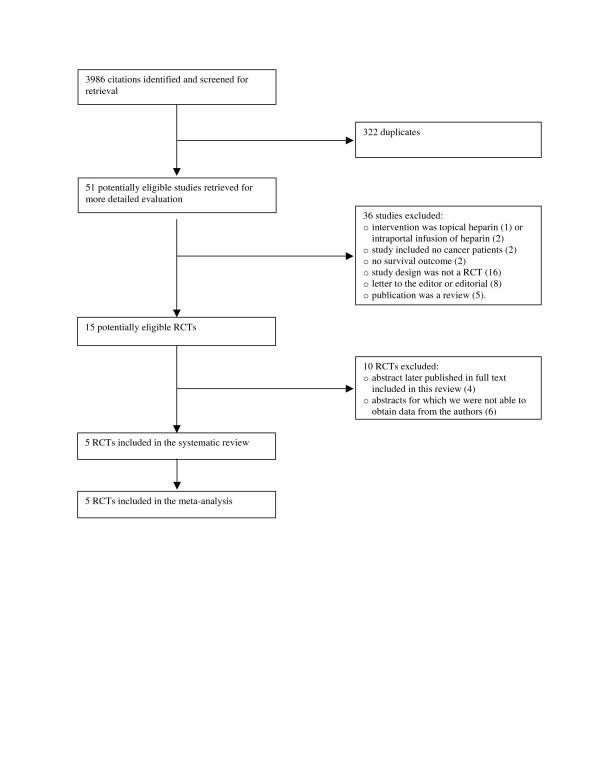
The trial flow.

Figure 1 shows the trial flow. The search strategy identified 3986 citations, including 322 duplicates. The title and abstract screening of the 3664 unique citations identified 51 as potentially eligible. The full text screening of the 51 citations identified 5 eligible RCTs published as full reports [6,7,12-14]. We identified 4 earlier published abstracts for 3 of the 5 included RCTs [12,15-17]. We also identified six eligible studies published as abstracts but we were unable to obtain the needed data from the authors [18-23]. Agreement between reviewers for eligibility was excellent (kappa = 0.94).

### Included studies

The five included studies recruited 1189 participants and reported follow-up data on 1175 [6-8,13,14]. The intervention was UFH in one study [13] and LMWH in four [6-8,14] and fondaparinux in none. Table 1 details the characteristics of these studies.

**Table 1:** Comparative table of randomized controlled trials assessing the effect of heparin on the survival of patients with cancer

**Study**	Methods*	**Intervention†**	**Participants‡**	**Outcome assessment§**	**Notes**
Lebeau 1994	AC: adequate Blinded: outcome assessors, data analyst ITT analysis: yes Study stopped early: no	UFH (prophylactic dose) vs. no intervention for 5 weeks; in combination with chemotherapy	Small cell lung cancer both limited and extensive; 78% had Karnofsky > 80; 277 randomized and 277 followed up (100%); 85% older than 50	Outcomes: mortality (at 12, 24, and 36 months)	Funding: None; maximum follow up: 84 months
Kakkar 2004 (FAMOUS trial)	AC: adequate Blinded: patients, care givers ITT analysis: yes Study stopped early: no	LMWH (Dalteparin; prophylactic dose) vs. placebo for 12 months; no restriction on concomitant chemotherapy or radiotherapy	Different types of with advanced stage III or IV malignant disease of the breast, lung, gastrointestinal tract, pancreas, liver, genitourinary tract, ovary, or uterus; minimum life expectancy 3 months; 385 randomized, 374 followed up (97%); no withdrawal from treatment; median age 61 IQR [53–68]	Outcomes: mortality (at 12, 24, and 36 months), PE, DVT, major bleeding, and minor bleeding Screening testing for DVT/PE: None Diagnostic testing for DVT/PE: not reported	Funding: Pharmacia Corp, NY; maximum follow up: 77 months
Klerk 2005 (MALT trial)	AC: adequate Blinded: patients, care givers, outcome assessors ITT analysis: yes Study stopped early: no	LMWH (Nadroparin) vs. placebo for 6 weeks; 2 weeks therapeutic dose then 4 weeks prophylactic dose; no concomitant chemotherapy or radiotherapy	Different types of solid malignant tumours "that could not be treated curatively" including: colorectal, breast, lung, gastric, oesophageal, liver, gallbladder, Katskin, prostate, pancreatic, cervical, urothelial, renal, ovarian, melanoma, endomaterial and other cancers; minimum life expectancy 1 month, stratified according to life expectancy (< or > 6 months); 302 patients randomized, 302 followed up (100%); median age 64	Outcomes: mortality (at 6, 12, and 24 months), major bleeding, and minor bleeding	Funding: Sanofi provided study medication; maximum follow up: 84 months
Altinbas 2004	AC: not reported Blinded:outcome assessors ITT analysis: yes Study stopped early: no	LMWH (Dalteparin; prophylactic dose) vs. placebo for 18 weeks or less if disease progressed; in combination with chemotherapy	Small cell lung cancer both limited and extensive, ECOG state < 3; 84 patients randomized, 84 patients followed up (100%); median age 58	Outcomes: mortality (at 12 and 24 months), DVT, and minor bleeding Screening and diagnostic testing for DVT: not reported	Funding: not reported; maximum follow up: 33 months
Sideras 2006	AC: adequate Blinded: patients, care givers, outcome assessors (1^st ^37% of randomized patients) ITT analysis: no Study stopped early for insufficient accrual	LMWH (Dalteparin; prophylactic dose) for unclear duration vs. placebo or no intervention	Different types of advanced cancer with minimum life expectancy 12 weeks; ECOG state 0–2; 141 randomized, 138 followed up (98%); no withdrawal from treatment; median age 67	Outcomes: mortality (at 12, 24, and 36 months), VTE, and major bleeding. Screening testing for DVT/PE: None Diagnostic testing for DVT: decided by the primary clinician	Funding: governmentally funded, pharmaceutical company supplied drug and placebo; maximum follow up: 24 months

* AC = allocation concealment; ITT = intention to treat analysis

† LMWH = Low molecular weight heparin; UFH = Unfractionated heparin

‡ ECOG = Eastern Cooperative Oncology Group

§DVT = deep venous thrombosis; PE = pulmonary embolism; VTE = venous thromboembolism

### Methodological quality of included studies

Allocation was adequately concealed in four studies [6,7,13,14] and it was unclear whether it was adequately concealed in the fifth study [8]. Two studies blinded participants, caregivers, and outcome assessors [7,14], one study blinded patients and caregivers [6], one study blinded outcome assessors and data analysts [13], and one study blinded only outcome assessors [8]. The lowest percentage of follow up in the five studies was 97%. Only one study did not use ITT analysis [14]. One study was stopped early for insufficient accrual [14]. According to GRADE methodology, the quality of evidence was moderate for survival, low for major and minor bleeding, and very low for DVT (Table 2).

**Table 2:** Summary of findings (SoF) table using GRADE methodology

**Parenteral anticoagulation for prolonging survival of patients with cancer**						
**Patient or population: **Patients with cancer						
**Settings: **Outpatient						
**Intervention: **Parenteral anticoagulation						

**Outcomes**	Illustrative comparative risks* (95% CI)		**Relative effect (95% CI)**	**No of Participants (studies)**	**Quality of the evidence (GRADE)**	**Comments**
					
	Assumed risk	Corresponding risk				

	**Control**	**Parenteral anticoagulation**				

**Survival**	**Low risk population**		**HR 0.77 **(0.65 to 0.91)	1174 (5)	⊕⊕⊕O **moderate**^4^	
					
	**500 per 1000**	**414 per 1000 **(363 to 468)				
					
	**Moderate risk population**					
					
	**1000 per 1000**	**1000 per 1000 **(1000 to 1000)				

**DVT**	**Low risk population**		**RR 0.61 **(0.08 to 4.91)	458 (2)	⊕OOO **very low**^1,2^	
					
	**10 per 1000**	**6 per 1000 **(1 to 49)				
					
	**High risk population**					
					
	**40 per 1000**	**24 per 1000 **(3 to 196)				

**Major bleeding**	**Low risk population**		**RR 1.50 **(0.26 to 8.8)	814 (3)	⊕⊕OO **low**^1,3^	
					
	**0 per 1000**	**0 per 1000 **(0 to 0)				
					
	**High risk population**					
					
	**100 per 1000**	**150 per 1000 **(26 to 880)				

**Minor bleeding**	**Low risk population**		**RR 2.07 **(0.78 to 5.51)	760 (3)	⊕⊕OO **low**^1,3^	
					
	**0 per 1000**	**0 per 1000 **(0 to 0)				
					
	**High risk population**					
					
	**30 per 1000**	**62 per 1000 **(23 to 165)				

*The basis for the **assumed risk **(e.g. the median control group risk across studies) is provided in footnotes. The **corresponding risk **(and its 95% confidence interval) is based on the assumed risk in the comparison group and the **relative effect **of the intervention (and its 95% CI).

CI: Confidence interval; RR: Risk ratio; HR: Hazard ratio;

GRADE Working Group grades of evidence

**High quality: **Further research is very unlikely to change our confidence in the estimate of effect.

**Moderate quality: **Further research is likely to have an important impact on our confidence in the estimate of effect and may change the estimate.

**Low quality: **Further research is very likely to have an important impact on our confidence in the estimate of effect and is likely to change the estimate.

**Very low quality: **We are very uncertain about the estimate.

^1 ^The 95% CI includes both negligible effect and appreciable benefit or appreciable harm

^2 ^Out of 5 included studies, only 2 reported DVT

^3 ^Out of 5 included studies, only 3 reported major bleeding

^4^Result statistically significant in only one subgroup.

### Quantitative results

**Figure 2:**
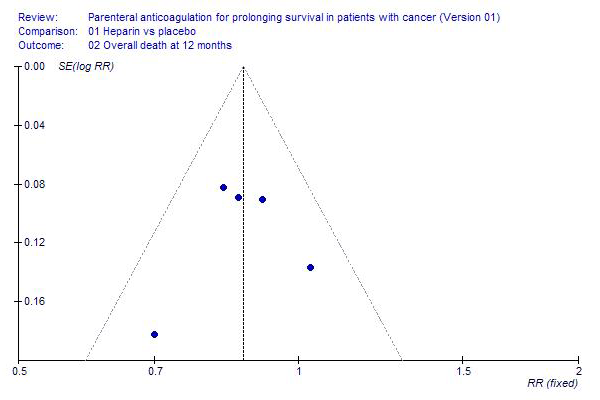
Inverted funnel plot for randomized controlled trials of parenteral anticoagulation in cancer patients.

There was low to moderate heterogeneity (I^2 ^= 47.5%) for the survival outcome. The small number of trials permitted subgroup analyses only for the subgroups of patients with small cell lung cancer (SCLC) and with "advanced cancer" (as defined in individual studies). The inverted funnel plot for the primary outcome of mortality at 1 year did not suggest publication bias (Figure 2).

#### All cause mortality

**Figure 3:**
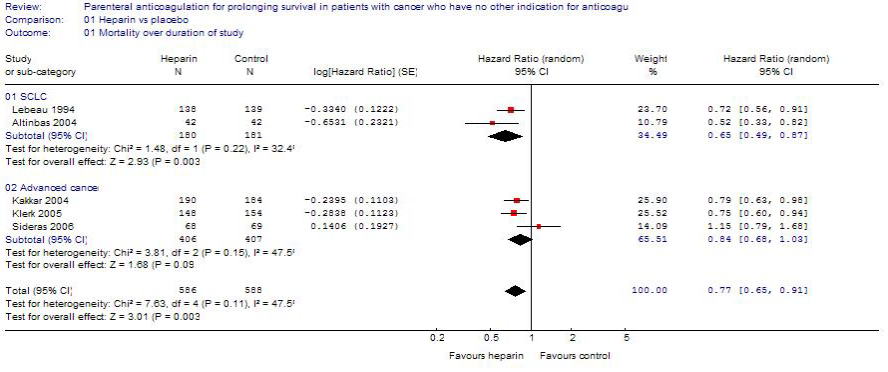
The effect of heparin therapy on survival in patients with cancer.

Based on a pooled estimate from the 5 RCTs, heparin was associated with a statistically significant survival benefit (HR = 0.77; 95%CI = 0.65 – 0.91; I^2 ^= 47%) (Figure 3). Excluding the study by Lebeau et al. [13] (the only study using UFH) then the study by Altinbas et al. [8] (in which the allocation was not clearly concealed) yielded estimates similar to the primary meta-analysis.

The categorical analysis confirmed those results with a statistically significant reduction of mortality at 12 months (RR = 0.87; 95%CI = 0.80–0.95) and at 24 months (RR = 0.92; 95%CI = 0.86 – 0.99).

#### Small cell lung cancer

**Figure 4:**
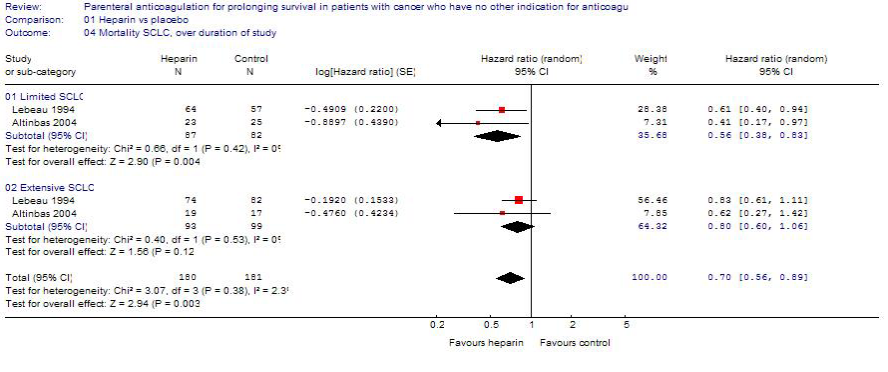
The effect of heparin therapy on survival in patients with small cell lung cancer.

In patients with limited SCLC, heparin was associated with a statistically significant survival benefit (HR = 0.56; 95%CI = 0.38 – 0.83), with no heterogeneity between trials (I^2 ^= 0) (Figure 4). In the categorical analysis, heparin was associated with a statistically significant reduction of mortality at 12 months (RR = 0.60; 95%CI = 0.42–0.87) but not at 24 months (RR = 0.90; 95%CI = 0.71–1.14). Excluding the study by Altinbas et al. did not change the results in terms of statistical significance.

For extensive SCLC, heparin was associated with a non-statistically significant survival benefit (HR = 0.80; 95%CI = 0.60–1.06; I^2 ^= 0) (Figure 4). The results were similarly non-statistically significant in the categorical analysis at 12 months (RR = 0.93; 95%CI = 0.76 = 1.15) and 24 months (RR = 0.88; 95%CI = 0.65–1.18).

#### Advanced cancer

Based on a pooled estimate from studies including patients with advanced cancer [6,7], heparin was associated with a non-statistically significant survival benefit (HR = 0.84; 95%CI = 0.68–1.03) (Figure 3), with moderate heterogeneity between trials (I^2 ^= 47%). The effect of heparin on mortality was borderline significant at 12 months (RR = 0.89; 95%CI = 0.80–1.00) and 24 months (RR = 0.92; 95%CI = 0.85–1.00).

Klerk et al [7] defined a priori two subgroups of patients with life expectancy less and greater than 6 months respectively. The HR for survival was 0.64 (95%CI = 0.45–0.90) for patients with longer life expectancy and 0.88 (95%CI = 0.62–1.25) for patients with shorter life expectancy)

#### Venous thromboembolism

Based on pooled estimates from two RCTs [6,8], heparin therapy was associated with a non-statistically significant reduction in DVT (RR = 0.61; 95%CI = 0.08–4.91).

#### Major and minor bleeding

**Figure 5:**
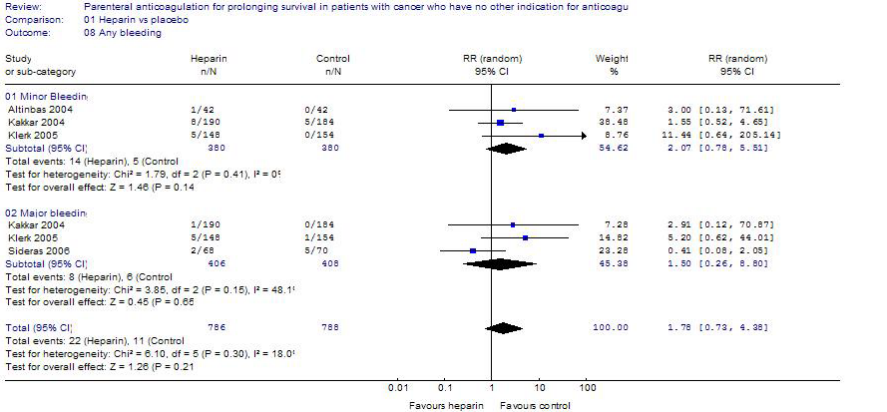
The effect of heparin therapy on bleeding in patients with cancer.

Pooled estimates showed that heparin therapy was associated with increased bleeding that was non-statistically significant for minor bleeding (RR = 2.07; 95%CI = 0.78–5.51), or major bleeding (RR = 1.50; 95%CI = 0.26–8.80) or any bleeding (RR = 1.78; 95%CI = 0.73–4.38) (Figure 5). After excluding the study by Altinbas et al. the results remained non-statistically significant.

Three studies assessed thrombocytopenia as an outcome but reported no events [7,8,13]. None of the studies reported participants withdrawing from treatment.

## Discussion

Heparin therapy (with either UFH or LMWH) was associated with a statistically and patient important survival benefit in cancer patients who had no indication for parenteral anticoagulation. In subgroup analyses, patients with limited SCLC experienced a clear survival benefit. The survival benefit was not statistically significant for either patients with extensive SCLC or patients with advanced cancer. The increased risk of bleeding with heparin was not statistically significant. We did not identify any study using fondaparinux as the anticoagulant.

The strengths of this study include our systematic approach to searching, study selection and data extraction which has minimized the likelihood of missing relevant studies. The quality of evidence was high for survival; all included studies were RCTs with moderate percentages of follow-up and allocation was clearly adequate in all but one included study. This moderate quality of evidence for surival, and the low likelihood of publication bias increase the confidence in the internal validity of our findings. Furthermore, we conducted pooled survival analysis for the important outcomes.

There was a statistically significant reduction in mortality at 12 months but not at 24 months in patients with limited SCLC. This difference probably reflects a true difference of effect at different follow-up periods. Such a difference might be due to the relatively short overall survival of patients with SCLC enabling short term but not long term benefit. It might also be due to the initial contribution of the antithrombotic effect of LMWH. While this assumption does not contradict a concomitant antitumor effect of LMWH (see below), it acknowledges the antithrombotic role and its clinical importance in managing patients with limited SCLC.

The non-significant findings in this study may be due to the small number of RCTs, of participants and of events. For example, compared with the data at 12 months, the results at 6 months tended to be non-significant; the latter could be explained by a smaller number of events in the early follow up period. The interpretation of findings is also limited by not including data from the 7 trials published as abstracts only.

Interpretation of the findings of this review is somewhat limited by the moderate heterogeneity between the results of different trials, which was not completely explained by subgroup analyses based on type of cancer. The heterogeneity could be related to variety in the stages of cancers, and in the types, dosing, schedules and duration of heparin therapy. The relatively small number of studies and the inclusion of different types of cancer in the same study precluded us from conducting the necessary subgroup analyses to explore all of these factors.

The statistically significant survival benefit of heparin in the subgroup of patients with limited SCLC in this review and in the subgroup of patients with life expectancy greater than 6 months in the study by Klerk et al [7] suggest that less ill patients receive greater benefit from heparin. The CLOT trial [24] supports these findings indirectly; in that study, patients with solid tumors and an acute venous thromboembolic event had improved survival if they did not have a metastatic disease at the time of study entry.

Studies with shorter periods of heparin therapy (i.e. 5 and 6 weeks) [7,13], appear to provide similar benefit as those with longer periods (i.e. 12 weeks) [6]. The clinical implication would be major if in fact, prolonging the duration of therapy does not provide additional benefit while increasing the risk of side effects (mainly bleeding events). However, none of the included studies was designed to address this question.

Lazo-Lannger et al. conducted a systematic review addressing the same question as this review [25]. Although that review had different inclusion criteria from our review (in particular, it excluded the trial of Lebeau) and obtained slightly different estimates of HRs using Parmar's methods, it reported similar results: a hazard rate comparing mortality in the heparin and control arms of 0.83 (95% CI: 0.70 to 0.99). This consistency of results from independent reviews confirms the robustness of the findings. However, Lazo-Lannger et al. did not report any subgroup analysis in patients with small cell lung cancer. While there are important pitfalls in subgroup analysis, they serve to generate hypotheses that should further be explored [26].

"The survival benefit in patients with cancer of anticoagulation is probably only in part mediated through an antithrombotic effect, i.e. through the prevention of fatal thromboembolic events. In fact, the survival curves of the included studies show consistent survival benefit beyond the duration of heparin therapy. Similarly, the meta-analysis shows a statistically significant survival benefit at 12 months while the duration of heparin therapy in 4 of the included studies was 5 weeks, 6 weeks, 18 weeks, and 12 months respectively. Experts in the field have attributed this phenomenon to an antitumor effect of anticoagulation [27,28].

Basic research supports the hypothesis of an antitumor effect of anticoagulation. Studies have implicated the tumour-mediated activation of the haemostatic system in both the formation of tumour stroma and in tumour metastasis [29-31]. There is also evidence that heparin inhibits expression of oncogenes, the formation by cancer cells of thrombin and fibrin induced, and the intravascular arrest of cancer cells, and thus metastasis [32].

The antitumor effect does not appear to be the same across anticoagulant classes. In a systematic review of oral anticoagulation for prolonging survival in patients with cancer, warfarin improved early survival in patients with extensive SCLC but not in patients with limited SCLC [33]. In another systematic review of the initial treatment of VTE in patients with cancer, LMWH provided a survival benefit compared with UFH [34]. Finally, for long term treatment of VTE in this population, LMWH compared with oral anticoagulation reduced the incidence of venous thromboembolism although did not provide a survival benefit [35].

## Conclusion

In conclusion, this systematic review suggests a survival benefit of heparin in cancer patients in general, and in patients with limited small cell lung cancer in particular. It also suggests a higher benefit in patients with limited cancer or a longer life expectancy. The decision for a patient with cancer to start heparin therapy for survival benefit should balance the benefits and downsides and integrate the patient's values and preferences [36]. Patients with a high preference for a short survival prolongation and limited aversion to bleeding who do not consider heparin therapy a burden may opt to use heparin, while those with aversion to bleeding may not.

Future research should investigate the effects anticoagulation in patients with different types and stages of cancers comparing different types, dosing and duration of therapy [37].

## List of abbreviations

DVT: Deep vein thrombosis; HR: Hazard ratio; ITT: Intention-to-treat; LMWH: low-molecular-weight-heparin; RCT: Randomized clinical trial; RR: Relative risk; SCLC: Small cell lung cancer; UFH: unfractionated heparin.

## Competing interests

Schünemann: no personal payments from for-profit sponsors, but he received research grants and honoraria that were deposited into research accounts or received by a research group that he belongs to from AstraZeneca, Amgen, Chiesi Foundation, Lily, and Pfizer, Roche and UnitedBioSource for development or consulting regarding quality of life instruments for chronic respiratory diseases and as lecture fees related to the methodology of evidence based practice guideline development and research methodology. Institutions or organizations that he is affiliated with likely receive funding from for-profit sponsors that are supporting infrastructure and research that may serve his work.

## Authors' contributions

EAA: protocol development, search for trials, screening, data extraction, data analysis, manuscript drafting, review coordination. MB: screening, data extraction. SR: screening, data extraction. IT: screening, data extraction. FS: screening, data extraction. PM: data analysis, methodological advice. HJS: protocol development, search for trials, data extraction, data analysis, methodological advice.

## Supplementary Material

Additional file 1 "Search strategies used for the electronic databases; parenteral anticoagulation to prolong survival systematic review". search strategy. Click here for file
