# Supplementary material for: Parenteral anticoagulation may prolong the survival of patients with limited small cell lung cancer: a Cochrane systematic review
Source: J Exp Clin Cancer Res. 2008 May 15;27(1):4. doi: 10.1186/1756-9966-27-4 (PMC2438335; doi:10.1186/1756-9966-27-4)
Supplement: Additional file 1 — "Search strategies used for the electronic databases; parenteral anticoagulation to prolong survival systematic review". search strategy. [file 1756-9966-27-4-S1.doc]

**Appendix:** Search strategies used for the electronic databases; parenteral anticoagulation to prolong survival systematic review

**MEDLINE**

#1 Heparin/

#2 Heparin.tw

#3 Heparin, Low-Molecular-Weight/

#4 (LMWH OR low molecular weight heparin OR nadroparin OR fraxiparin OR enoxaparin OR clexane OR lovenox OR dalteparin OR fragmin OR ardeparin OR normiflo OR tinzaparin OR logiparin OR innohep OR certoparin OR sandoparin OR reviparin OR clivarin OR danaproid OR orgaran).tw

#5 1 OR 2 OR 3 OR 4

#6 Coumarins/

#7 Warfarin/

#8 (warfarin OR coumadin OR acenocumarol OR phenprocumon OR 4-hydroxicoumarins OR oral anticoagulant OR vitamin K antagonist OR VKA).tw

#9 6 OR 7 OR 8

#10 (fondaparinux OR Arixtra).tw

#11 (ximelagatran OR Exanta).tw

#12 5 OR 9 OR 10 OR 11

#13 Neoplasms/

#14 (malignan$ OR neoplasm$ OR cancer OR carcinoma$ OR adenocarcinoma OR tumour OR tumor).tw

#15 13 OR 14

#16 clinical trial.pt. OR random:.tw. OR tu.xs.

#17 animals/ NOT human/

#18 16 NOT 17

#25 12 AND 15 AND 18

**EMBASE**

#1 Heparin/

#2 heparin.tw

#3 Low Molecular Weight Heparin/

#4 (LMWH OR low molecular weight heparin OR nadroparin OR fraxiparin OR enoxaparin OR clexane OR lovenox OR dalteparin OR fragmin OR ardeparin OR normiflo OR tinzaparin OR logiparin OR innohep OR certoparin OR sandoparin OR reviparin OR clivarin OR danaproid OR orgaran).tw

#5 1 OR 2 OR 3 OR 4

#6 Coumarin derivative/

#7 Warfarin/

#8 (warfarin OR coumadin OR acenocumarol OR phenprocumon OR 4-hydroxicoumarins OR oral anticoagulant OR vitamin K antagonist OR VKA).tw

#9 6 OR 7 OR 8

#10 fondaparinux/

#11 (fondaparinux OR Arixtra).tw

#12 ximelagatran/

#13 (ximelagatran OR Exanta).tw

#14 5 OR 9 OR 10 OR 11 OR 12 OR 13

#15 Neoplasm/

#16 (malignan$ OR neoplasm$ OR cancer OR carcinoma$ OR adenocarcinoma OR tumour OR tumor).tw

#17 15 OR 16

#18 Random:.tw. OR clinical trial:.mp. OR exp health care quality

#19 animals/ NOT human/

#20 18 NOT 19

#21 14 AND 17 AND 20

**ISI (International Scientific Information) the Web of Science**

#1 heparin OR low molecular weight heparin OR LMWH OR low-molecular-weight-heparin OR nadroparin OR fraxiparin OR enoxaparin OR clexane OR lovenox OR dalteparin OR fragmin OR ardeparin OR normiflo OR tinzaparin OR logiparin OR innohep OR certoparin OR sandoparin OR reviparin OR clivarin OR danaproid OR orgaran

#2 Coumarins OR Warfarin OR coumadin OR acenocumarol OR phenprocumon OR 4-hydroxicoumarins OR oral anticoagulant OR vitamin K antagonist OR VKA

#3 fondaparinux OR Arixtra

#4 ximelagatran OR Exanta

#5 1 OR 2 OR 3 OR 4

#6 malignan$ OR neoplasm$ OR cancer OR carcinoma$ OR adenocarcinoma OR tumour OR tumor

#7 random$ OR placebo$ OR versus OR vs OR double blind OR double-blind OR compar$ OR controlled

#8 5 AND 6 AND 7

**CENTRAL (The Cochrane Library, latest issue)**

#1 heparin OR low molecular weight heparin OR LMWH OR low-molecular-weight-heparin OR nadroparin OR fraxiparin OR enoxaparin OR clexane OR lovenox OR dalteparin OR fragmin OR ardeparin OR normiflo OR tinzaparin OR logiparin OR innohep OR certoparin OR sandoparin OR reviparin OR clivarin OR danaproid OR orgaran

#2 Coumarins OR Warfarin OR coumadin OR acenocumarol OR phenprocumon OR 4-hydroxicoumarins OR oral anticoagulant OR vitamin K antagonist OR VKA

#3 fondaparinux OR Arixtra

#4 ximelagatran OR Exanta

#5 1 OR 2 OR 3 OR 4

#6 malignan$ OR neoplasm$ OR cancer OR carcinoma$ OR adenocarcinoma OR tumour OR tumor

#7 5 AND 6
